# Detection probabilities of bird carcasses along sandy beaches and marsh edges in the northern Gulf of Mexico

**DOI:** 10.1007/s10661-019-7924-z

**Published:** 2020-03-17

**Authors:** Guthrie S. Zimmerman, Veronica W. Varela, Julie L. Yee

**Affiliations:** 1 U. S. Fish and Wildlife Service, Division of Migratory Bird Management, 3020 State University Drive East, Modoc Hall, Suite 2007, Sacramento, CA 95819 USA; 2 U.S. Fish and Wildlife Service, Natural Resource Damage Assessment and Restoration Program, 1011 E Tudor Rd, MS 361, Anchorage, AK 99503 USA; 3 U. S. Geological Survey, Western Ecological Research Center, 2885 Mission Street, Santa Cruz, CA 95060 USA

**Keywords:** Carcass detection, Gulf of Mexico, Marsh edge, Oil spill, Sandy beach, Detection probability

## Abstract

We estimated detection probabilities of bird carcasses along sandy beaches and in marsh edge habitats in the northern Gulf of Mexico to help inform models of bird mortality associated with the *Deepwater Horizon* oil spill. We also explored factors that may influence detection probability, such as carcass size, amount of scavenging, location on the beach, habitat type, and distance into the marsh. Detection probability for medium-sized carcasses (200–500 g) ranged from 0.82 (SE = 0.09) to 0.93 (SE = 0.04) along sandy beaches. Within sandy beaches, we found that intact/slightly scavenged carcasses were easier to detect than heavily scavenged ones and did not find strong effects of location on the beach on detection probability. We estimated detection rate for each combination of scavenging state, carcass size, and position along sandy beaches. In marsh edge habitats, detection ranged from 0.04 (SE = 0.04) to 0.86 (SE = 0.10), with detection rates rapidly increasing from small (< 200 g) to medium carcass sizes and leveling off between medium and extra-large (> 1000 g) carcasses regardless of vegetation type (*Spartina* or *Phragmites*). Carcasses of all sizes were generally harder to locate in *Spartina*-dominated marshes than in *Phragmites*-dominated ones. A subset of the data for which we could adequately assess the effect of distance into the marsh indicated that detection rates generally declined the farther a carcass was into marsh vegetation. Based on power analyses, our ability to identify predictors that influence detection rates would be higher with larger numbers of carcasses, greater numbers of search trials per carcass, or more balanced sampling distributions across predictor values.

**Electronic supplementary material:**

The online version of this article (10.1007/s10661-019-7924-z) contains supplementary material, which is available to authorized users.

## Introduction

The number of bird carcasses deposited along beaches is used as an index to quantify mortality from oil spills (Ford 2006; Ford et al. 1996, 2001, 2009). Consequently, biologists commonly implement beached bird surveys following oil spills to count the number of, and collect, carcasses found along beaches in the vicinity of oil spills. These counts represent underestimates of the bird carcasses deposited on the shore because observers do not detect every carcass that is present and visible (e.g., perception bias; Marsh and Sinclair 1989), and other animals scavenge and remove carcasses or carcasses are covered by debris and sand making it impossible for observers to detect them (e.g., availability bias; Marsh and Sinclair 1989). Consequently, mathematical models are commonly used during natural resource damage assessment (NRDA) to adjust the number of carcasses collected along shorelines for the efficiency of searchers in finding carcasses that are available for detection (hereafter, “detection probability”) and the rate that scavengers and other activities remove carcasses from shorelines before searchers can find them (i.e., carcass persistence).

On April 20, 2010, an explosion and subsequent fire on the *Deepwater Horizon* oil rig led to the sinking of the rig and an uncontained oil leak for 87 days (hereafter “DWH”). The specific source of the DWH leak was the Macondo Prospect (Mississippi Canyon Block 252) in the northern Gulf of Mexico. The approximately 134 million gallons of oil that escaped during the 87 days eventually reached the shorelines from eastern Texas to the panhandle of Florida. A collaboration of state and federal governmental agencies formed the Natural Resource Damage Assessment and Restoration Trustee Council (hereafter “Trustees”) and conducted a comprehensive natural resource damage assessment to quantify the harm done by the DWH oil spill to natural resources and identify necessary restoration activities. As part of the process to quantify harm to birds, the Trustees counted and collected bird carcasses along shorelines within and adjacent to the spill and implemented field studies to improve overall estimates of the total number of carcasses deposited on shorelines based on a shoreline deposition model (e.g., Amend et al. 2020). An important input for the model was the detection rate of bird carcasses, assuming they were available to be seen, by search teams surveying shorelines.

Previous research has indicated that detection rates of carcasses are variable and are influenced by a variety of factors (Fowler and Flint 1997; Morrison 2002; Ford 2006; Byrd et al. 2009). Consequently, understanding detection rates under local conditions is critical for improving estimates of abundance of carcasses during an oil spill in a particular region. Several methods are available to estimate or account for detection rates (Nichols 1992; Williams et al. 2001; Nichols et al. 2009) and each has associated assumptions and weaknesses depending on objectives and local conditions (Johnson 2008; Nichols et al. 2009). To estimate detection rates during beached bird monitoring along the northern Gulf of Mexico following the DWH oil spill, Trustees implemented field studies by placing bird carcasses at known locations along sandy beach and marsh shoreline segments and observed how many of the carcasses were detected by search teams. The field studies were designed and implemented by staff from the U.S. Fish and Wildlife Service and the State of Louisiana Department of Wildlife and Fisheries, on behalf of the greater group of Natural Resource Trustees for the *Deepwater Horizon* NRDA, with contracted assistance from Industrial Economics, Inc. (Cambridge, MA) and R.G. Ford Consulting, Inc. (Portland, Oregon) (hereafter “field investigators”). Previous research indicated that detection likely varies by carcass size, so the primary objective of our analysis was to use mark-recapture techniques to derive estimates of detection for different sized carcasses in the northern Gulf of Mexico. During field sampling, field investigators noted additional factors that may influence detection rates, such as carcass state (i.e., condition of the carcass with respect to scavenging), carcass location on the beach, habitat type, and distance into marsh vegetation from the vegetation/open water interface. Therefore, our secondary objective was to explore the potential influence of these additional factors on detection rates to the extent possible given available data. In cases where sample sizes were of questionable adequacy for testing additional factors, our additional objective was to examine statistical power, i.e., probability of finding significant factors when they exist, and study design elements that can improve power.

## Materials and methods

### Bird carcasses

No birds were killed for the purposes of implementing the detection probability studies. Most of the study carcasses were salvaged from various legally permitted animal control programs throughout the USA that collected birds using firearms with non-toxic shot or were captured alive and humanely euthanized. None of the study carcasses was affiliated with oil spills or disease-related mortality events. A minority of the carcasses were provided by natural resource management agencies that had incidentally collected specimens in good condition through routine management activities. Thus, the relative proportions of bird sizes for these studies depended on what investigators could collect from these sources, and samples distributed across size classes were unbalanced. Birds were stored frozen until prepared for use in the studies. Carcasses were classified into four size categories: small (< 200 g), medium (200–500 g), large (501–1000 g), and extra-large (> 1000 g).

### Site description and field methods

#### Sandy Beach

Field investigators distributed 128 carcasses over 39 sandy walkable transects, each 2 km in length, between Sabine Pass (Texas-Louisiana border) and Panama City, FL, in September 2010. Study carcasses were not oiled and consisted primarily of various species of gulls. Each carcass was randomly assigned a specific location along the transect and position on the beach relative to the high tide line (i.e., below, at, or above the wrack line). Field investigators did not have access to equal numbers of carcasses for each size class and no large category carcasses were available. Consequently, the available sample data for the detection study resulted in an unbalanced sample among size classes with 8% small, 69% medium, 0% large, and 23% extra-large (Donlan et al. 2013a). During the oil spill incident, field investigators noted that 31.6% of collected carcasses were heavily scavenged (internal organs and pectoral muscles removed), skeletal remains, or in mummified conditions (Industrial Economics, Inc. (IEc) 2011). The field investigators attempted to mimic variations in scavenging state in case they influence detection. Heavily scavenged study carcasses were created by removing the internal organs, but, given the study schedule, they were not able to create skeletal remains or mummified conditions. Thus, they used heavily scavenged carcasses to represent all carcass condition categories other than “intact/lightly scavenged.” Twenty-nine percent of the study carcasses were treated to be heavily scavenged. Because the goals of the study had been to estimate size-specific detection rates, the field investigators did not optimize the sampling distribution among size classes, positions on the beach, and scavenging states to test differences among the three variables.

The study was designed to test the detection ability of the actual personnel that conducted the searches for spill-related bird carcasses during the oil spill event. Thus, the study was implemented while these searches were still ongoing. Field investigators placed study carcasses along study transects a few hours before search teams conducted their normal surveys for beached carcasses. Deployment teams tried to minimize their tracks and attempted to make study carcasses look natural. Each study carcass was marked with a numbered plastic ring and a small laminated card. The card was positioned underneath the study carcass to avoid drawing additional attention. The card displayed an orange circle and identified the carcass as part of a “tagged carcass study.” The cards helped to ensure that study carcasses were not accidentally collected as spill-victim birds. Eight search teams consisting of two observers were tested, and each transect was searched once by a single search team. Search teams were not fully aware that they were participating in an assessment of detection probabilities. However, they were informed that if they observed a carcass with an attached laminated card with an orange dot on it, they should note the tagged carcass on the back of their datasheets and leave the carcass in place. Field investigators noted whether the search team found each planted carcass, carcass size and scavenging state, carcass position on the beach relative to the tide line, and search team. Searches were conducted as one pass in one direction through the transect (i.e., not as an out-and-back search path). After search teams completed their surveys, the study carcasses were retrieved later that day.

#### Marsh edge

The marsh edge detection probability study was conducted along estuarine marshes in Louisiana in late October and early November, 2011 (Trustee Bird Technical Working Group 2012; Donlan et al. 2013b). The methods used to investigate marsh edge searcher detection were similar to the methods described for sandy beach detection probability, in that field investigators placed carcasses in known locations for searchers to discover. However, there were five important differences in the marsh edge study design: (1) there were three discrete search teams that surveyed each of the study transects once, attempting to find the same set of carcasses deployed in each transect; (2) search teams looked for carcasses from boats rather than walking shorelines; (3) the majority of carcasses appeared intact/slightly scavenged at deployment (6.9% of deployed carcasses showed some signs of early decay); (4) four carcass size categories were used in the field (small, medium, large, and extra-large); and (5) search teams were aware that they were participating in a detection probability study effort, although they had no a priori knowledge of specific carcass locations. Trustee agency personnel that conducted searches in 2010 for carcasses associated with DWH spill were recalled to comprise the search teams in this study.

Field investigators deployed 87 carcasses along 17 1-km transects for three discrete teams to independently search by boat along marsh edges. Study carcasses were comprised of 20 species, with the majority being various species of gulls. All carcasses were intact/slightly scavenged, non-oiled birds. The distribution of carcass sizes depended upon the carcasses available for use but was purposefully biased toward medium-sized carcasses after considering feedback from interviews of spill response personnel that collected DWH-related carcasses from marsh edges during the spill, the majority of which were medium-sized birds. The primary goal of the study had been to estimate size-specific detection rates, but study carcasses were purposefully deployed in two marsh types (*Spartina-* or *Phragmites*-dominated habitats) and at varying distances into the marsh, in case those factors influenced detection. Sampling distribution among size classes and habitat types was not optimized to test differences between these two variables, as this was not the original intention of the field study.

Each study carcass was marked with numbered plastic rings and a small laminated card attached to the bird identifying the carcass as part of a study. Deployment locations were watery environments with emergent vegetation. In some cases, to keep study carcasses from drifting away during the search trials, the carcasses were lightly tethered to nearby vegetation by thread. For birds deployed further into marsh vegetation, birds were placed with long poles from boats so that vegetation was not matted and no visual cues for bird locations occurred. Each search team consisted of two persons, and three search teams were given separate opportunities to search each transect. Each search was comprised of one pass along a transect. After all three search teams completed their search trials at a transect (2 to 6 h after deployment), the field investigators verified the presence and locations of all 87 of the study carcasses to confirm that each carcass was indeed present for each search team to potentially find. Field technicians recorded the number of carcasses each search team found on each transect, distance of the carcass into the marsh vegetation, carcass species and size, and marsh type along the transect.

### Statistical analysis

We modeled carcass detections in relationship to predictor variables (hereafter “predictors”) that we hypothesized to potentially influence detection probability, e.g., carcass size and habitat. Specifically, we used generalized linear models with a binomial distribution to model the number of trials (i.e., searches) in which a carcass is detected, from the total number of trials, to estimate detection probability and the effects of its predictors (Neter et al. 1996). We expected that the largest carcasses would be the easiest to detect and, conversely, the smallest carcasses the most difficult.

We used two criteria for assessing the influence of predictors on the detection of carcasses: (1) information criteria, which can be used to compare and rank different models and therefore identify model predictors or combinations of predictors useful for estimating detection probability and (2) parameter estimates, which indicate the strength and direction (positive or negative) of the relationship between predictors and detection probability while accounting for the influence that other predictors in the model may have on detection probability. We used Akaike’s Information Criteria corrected for small sample size (AIC_*c*_), or quasi-AIC_*c*_ (QAIC_*c*_) if replicate searches indicated overdispersion in the data. The model with the lowest AIC_*c*_ or QAIC_*c*_ value was considered the most parsimonious or “best” model (Burnham and Anderson 1998). We assessed individual predictors within models by examining whether the 95% confidence intervals (CI) for the parameter estimates overlapped zero. A parameter estimate greater than zero with CI that excludes zero indicates a positive correlation between the predictor and detection probability, whereas a parameter estimate less than zero with CI that excludes zero indicates a negative correlation, and a parameter estimate of zero indicates we could not detect a correlation with our data. We used the “generalized linear model” (glm) function in program R (R Core Team 2012) for conducting these analyses.

In addition to exploring model-specific parameter estimates, we estimated detection probability across a range of predictor values and computed weighted averages of these estimates across all models, i.e., model-averaged estimates, using AIC_*c*_ weights to accommodate model selection uncertainty (Burnham and Anderson 1998). If a model failed to adequately converge as evidenced by inflated standard errors (> 1000), as would occur when attempting to model probabilities of perfect 1 or 0 (i.e., complete successes or failures within a predictor class or combination of classes, also known as separation), then we excluded that model from contributing to model-averaged estimates of detection (Heinze 2006). We used the data analysis package “AICcmodavg” (Mazerolle 2013) to calculate model-averaged detection estimates in program R (R Core Team 2012). Although we made every attempt to analyze the sandy beach and marsh edge studies similarly, there were additional considerations specific to each study described separately below.

#### Sandy Beach

For the sandy beach study, in which field investigators conducted only one search trial per carcass, the response variable was 1 if bird was found or 0 if not. Predictors included location of the carcass relative to the surf and scavenging state of the carcass. There were no large carcasses, and the small number of available small and extra-large carcasses resulted in several instances of only 0 or 1 carcasses in some combinations of predictors (e.g., we had no samples of small carcasses in the heavily scavenged state along the lower beach). Therefore, we limited the analysis of the sandy beach data to the medium-size category (88 of 128 carcasses), which was the only one with > 5 carcasses in every combination of scavenging state and position. We predicted that birds lower on the beach would be the most detectable due to the smooth, homogeneous surface of the beach that often occurs below the most recent high tide line, but we were unsure whether birds in the middle of the beach and associated with the wrack would be more or less detectable than those above the wrack and the high tide line. Therefore, we considered position to be a categorical predictor with these three position levels. We also considered the scavenging state of the carcasses when deployed as a categorical predictor. Although the field search teams generally did not record the scavenging state of carcasses they found during the study, each study carcass was deployed in the field for less than 12 h, and thus, additional natural scavenging or decomposition during deployment was presumed to be minimal. We considered various combinations of the two predictors in single-parameter, additive, and interaction models (Table 1).

**Table 1:** Model selection results for study assessing factors that may affect detection of medium-sized (200–500 g) bird carcasses along sandy beaches. *State* is the state of the carcass (heavily scavenged, or intact/lightly scavenged). *Position* is the categorical variable for position of the carcass on the beach (seaward of the wrack, at the wrack line, and landward of the wrack)

Model^a^	Number of parameters	AIC_*c*_	Difference in AIC_*c*_	AIC_*c*_ weight	Log likelihood
State	2	62.63	0.00	0.61	− 29.24
Intercept only	1	64.36	1.73	0.26	− 31.16
State + position	4	66.34	3.71	0.10	− 28.93
Position	3	68.00	5.37	0.04	− 30.86
State × position ^b^	6	–	–	–	–

^a^“+” represents additive effects and “×” represents interaction effects

^b^Model did not converge

#### Marsh edge

In the marsh edge study, the number of search trials conducted per carcass was equal to the number of search teams (except for one carcass that only produced two trials of usable data), and the response variable was the number of trials in which the carcass was detected. Predictors included carcass size, marsh type, and distance into marsh. Because multiple trials per carcass were conducted, we were able to estimate overdispersion in the data. Overdispersion arises frequently in observational environmental studies because the variance of count data often exceeds theoretical expectations, e.g., binomial variance functions formed from detection probability, after all sources of variation have been taken into account (Gelman and Hill 2007). Such overdispersion does not lead to biased estimates, but can lead to underestimated variances and overstated precision and confidence if not properly adjusted for (McCullagh and Nelder 1989). The calculation of QAIC_*c*_ incorporates overdispersion directly and is characterized by an increased model selection uncertainty compared with AIC_*c*_ (Burnham and Anderson 1998). We calculated an overdispersion factor ($\hat{c}$) by fitting a global model containing all predictors, taking the sum of its squared Pearson residuals, and dividing by the residual degrees of freedom (Faraway 2006). A $\hat{c}$ of 1 indicates no overdispersion, whereas $\hat{c}$ > 1 indicates overdispersed data (Faraway 2006).

Unlike with the sandy beach study, we did not evaluate scavenging state of the carcass, as all study carcasses were in relatively intact condition. We also did not model the influence of search team on detection because data summaries of the multiple trials indicated that all teams tended to detect the same carcasses (see results). We modeled two of the predictors, carcass size and habitat type, by themselves and in various combinations of additive and interaction models (see Table 2 for a list of all models considered). We expected that the largest carcasses would be the easiest to detect and, conversely, the smallest carcasses the most difficult. However, we could not assume a linear relationship between carcass size and detection, so we included size as a categorical predictor. We did not have an a priori prediction about how detection rates would compare between the two marsh habitats. We used the glm function in program R (R Core Team 2012) for conducting these analyses and also used model averaging for presenting fitted values of detection for different carcass sizes and habitat types.

**Table 2:** Model selection results for study assessing factors that may affect detection of bird carcasses along marsh edges. *Size* is the size of carcass (small [< 200 g], medium [200 to 500 g], large [501 to 1000 g], and extra-large [> 1000 g]). *Habitat* is the different marsh types (*Phragmites*- or *Spartina*-dominated)

Model^a^	Number of parameters	QAIC_*c*_^b^	Difference in QAIC_*c*_	QAIC_*c*_ Weight	Log likelihood
Size + habitat	6	114.29	0	0.98	− 50.59
Size	5	122.53	8.24	0.02	− 55.87
Habitat	3	127.36	13.07	0.00	− 60.53
Intercept only	2	132.69	18.40	0.00	− 64.27
Size × habitat^c^	9	–	–	–	–

^a^“+” represents additive effects; and “×” represents interaction effects

^b^Overdispersion = 2.36

^c^Model did not converge

Because of unbalanced and low sample sizes (< 5 carcasses for some combinations of carcass size and habitat categories) in the marsh edge study design, we were uncertain of its power to reasonably detect carcass size, habitat, and their interaction effects. We conducted simulation analyses to estimate power under hypothetical study conditions based on the binomial model for the marsh edge study except with different numbers of search trials per carcass (1 through 6), doubling of the number of carcasses, and a balanced distribution of carcasses (e.g., 10 or 20 carcasses per each of the 8 combinations of size class and habitat categories). We simulated data by randomly drawing search trial outcomes from binomial distributions according to a generalized linear model with a continuous size covariate (1 = small, 2 = medium, 3 = large, 4 = extra-large) and model parameters that describe a detection rate which increases with size and is lower for one habitat than the other (*β*_intercept_ = − 1, *β*_size_ = 1, *β*_habitat_ = − 2). We repeated simulations with and without an interaction effect describing mitigated differences between the two habitats as carcass size increases (*β*_size × habitat_ = 0.5), i.e., where habitat differences are greater for small carcasses than for large carcasses. Also, because Pearson estimates of overdispersion can potentially be biased if the distribution of residuals are not chi-square distributed, we simulated data with a prescribed overdispersion parameter of 2.35 using an individual heterogeneity model based on a beta-binomial distribution and recalculated Pearson values to assess whether $\hat{c}$ estimates were generally accurate, i.e., close to 2.35, or biased. We repeated dataset simulations for 10,000 iterations, and fit binomial models with size, habitat, and interaction effects to each dataset. For each effect, we present the mean and SD of the $\hat{c}$ estimates from the simulations and calculated power as the proportion of datasets which resulted in a statistically significant test at alpha = 0.05.

Lastly, we explored the potential influence of distance into the marsh on detection probabilities. Although specifying distance into a marsh seems straightforward, most marshes did not have a clear boundary that marked the edge of the marsh and the start of the ocean. Because of difficulties in measuring distance due to this issue, we conducted this analysis to gain a cursory understanding of how distance may influence detection. Further, the end-of-trial checks revealed that six carcasses had disappeared at some unknown point during the study, another six carcasses were found in locations other than the deployment locations (mean movement = 0.8 m, range = 0.15–1.7 m), and yet another six carcasses did not have complete distance information to allow an analysis of movement. Thus, we evaluated the effects of distance into marsh on a subset of the marsh edge study data, focusing on those carcasses which we were confident did not move from their deployment locations during the study (69 carcasses). Because not all carcasses had distance information, the sample size for assessing the influence of distance on detection probability was smaller than the sample used to explore just the potential influences of size and marsh type and the results for the two analyses are not directly comparable (Burnham and Anderson 1998). The relative influence of distance into the marsh can vary by marsh habitat type and carcass size, so we attempted to control for these variables when exploring the influence of distance on detection rates. However, the sample size of carcasses in the *Phragmites* marshes was small for each size class (4 small, 6 medium, 1 large, and 3 extra-large) after removing the carcasses that moved or had missing distance data. During the implementation of the study, the ranges of the distances at which carcasses were deployed were slightly different between marsh habitat types, which could confound the effect of distance into marsh if habitat type was left unaccounted. Deployment distances into *Spartina* ranged from zero to 2.85 m (mean = 0.79 m), while distances into *Phragmites* ranged from zero to 2.25 m (mean = 0.42). Because of the small sample of carcasses in *Phragmites* and the potential confounding due to different distances in the two marsh habitats, we limited the analysis of distance into the marsh to the samples in the *Spartina* marshes. The distance into the marsh (in m) was a continuous predictor, and carcass size was analyzed as a categorical predictor. We considered various combinations of distance and size class in single-parameter, additive, and interaction models (Table 3). We used the glm function in program R (R Core Team 2012) for conducting the analysis of distance. We calculated $\hat{c}$ as described above to account for overdispersion.

**Table 3:** Model selection results for a study assessing the influence of distance into marsh on the detection of bird carcasses in *Spartina*-dominated marshes. *Distance* is the continuous measure of distance into marsh from the open water-marsh boundary (in m). *Size* is the carcass size class (1 = small [< 200 g], 2 = medium [200 to 500 g], 3 = large [501 to 1000 g], and 4 = extra-large [> 1000 g])

Model^a^	Number of parameters	QAIC_*c*_^b^	Difference in QAIC_*c*_	QAIC_*c*_ weight	Quasi-log likelihood
Distance + size	6	72.20	0.00	1.00	− 29.22
Distance	3	99.82	27.62	0.00	− 46.67
Size	5	109.31	37.12	0.00	− 49.04
Intercept only	2	129.25	57.05	0.00	− 62.51
Distance × size^c^	9	–	–	–	–

^a^“+” represents additive effects; and “×” represents interaction effects

^b^Overdispersion = 1.58

^c^Model did not converge

## Results

### Sandy beach

A total of 78 carcasses were detected out of the 88 medium-sized carcasses that were distributed. The best model for detection probability included carcass state as a predictor, but not position (AIC_*c*_ weight = 0.61; Table 1). Specifically, parameter estimates from this model indicated that intact/slightly scavenged bird carcasses had higher detection rates than heavily scavenged carcasses (*β*_state_ = 1.34, SE = 0.69; where intact/slightly scavenged carcasses = 1 and heavily scavenged carcasses = 0). The intercept-only model ranked second best with an AIC_*c*_ difference of 1.73, which indicates that a simpler model with no effects was competitive with the best model. The position of the carcass on the beach did not appear to be important, because including that parameter did not improve model fit, and confidence intervals for the position predictor included zero in all models containing that variable, which indicates no or a very weak correlation. The interaction model between carcass state and position did not converge, likely due to the fact that all intact/slightly scavenged carcasses in the wrack and upper beach were detected. Model-averaged estimates of detection probability ranged from 0.82 (SE = 0.09) for medium-sized heavily scavenged carcasses along the upper beach to 0.93 (SE = 0.04) for intact/slightly scavenged medium-sized carcasses along wrack (Table 4).

**Table 4:** Model-averaged estimates of detection rates for medium-sized carcasses (200 to 500 g) in two scavenging states along sandy beaches

State		*N*^a^	Estimate	Standard error	% Coefficient of variation
Heavily scavenged	Lower beach	9	0.83	0.09	10
Heavily scavenged	Wrack	6	0.83	0.09	11
Heavily scavenged	Upper beach	13	0.82	0.09	11
Intact/lightly scavenged	Lower beach	9	0.92	0.04	5
Intact/lightly scavenged	Wrack	20	0.93	0.04	4
Intact/lightly scavenged	Upper beach	31	0.92	0.04	5

^a^Total number of carcasses deployed in each scavenging state and position category

### Marsh edge

Eighty-seven carcasses were distributed in marshes along the coast, of which 81 were confirmed by post-study checks as having been present and available for all searchers to detect during the study. In addition, one carcass was found by two teams during the study but disappeared by the time that post-study checks were conducted; thus, we cannot be sure that one carcass was available to be found by the third search team. Considering these disappearances, a total of 245 searches were evaluated (81 × 3 + 1 × 2 = 245). The search teams had almost identical numbers of detections among them (team 1 = 33, team 2 = 34, and team 3 = 33), and the composition of size classes and habitats of detected carcasses were similar among the search teams. Further, the search teams tended to consistently find or miss the same carcasses (84% of the carcasses were either missed by all three search teams or detected by all three search teams). As a result of this low variability among search teams, we did not include search team effect in models.

The overdispersion estimate from our observed data was 2.36. Following 10,000 simulations of data with this overdispersion and correspondingly recalculating overdispersion, and averaging across study conditions, we observed a median overdispersion estimate of 2.30 (mean = 2.30, SD = 0.26), so the method we used for estimating overdispersion did not appear to be greatly biased. We adjusted for overdispersion by scaling the variances of parameter estimates by this factor to make inferences regarding estimates and model selection (QAIC_*c*_). The QAIC_*c*_ values indicated that detection of carcasses varied by size class and habitat (Table 2). The best model included additive effects of size and habitat and indicated that larger birds had higher detection rates than smaller ones regardless of habitat (*β*_medium_ = 2.55, SE = 0.93; *β*_large_ = 3.15, SE = 0.98; *β*_extra-large_ = 3.20, SE = 0.98). Overall, carcasses were harder to locate in *Spartina*-dominated marshes than in *Phragmites*-dominated ones (*β*_habitat_ = − 1.89, SE = 0.64; where *Phragmites* = 0 and *Spartina* = 1). The model with an interaction between marsh type and size effects did not converge, due to all extra-large carcasses in *Phragmites* being detected by all three teams. Model-averaged estimates of detection probability along marsh edges were lower than those along sandy beaches and ranged from 0.04 (SE = 0.04) for small carcasses in *Spartina*-dominated marshes to 0.86 (SE = 0.10) for extra-large carcasses in *Phragmites*-dominated marshes (Table 5). These model-averaged estimates illustrate that the detection rates appeared to increase rapidly from small- to medium-sized carcasses but were similar among medium, large, and extra-large carcasses regardless of marsh type (Table 5).

**Table 5:** Model-averaged estimates of detection rates and unconditional standard errors^a^ for carcasses in two marsh types and four carcass size categories

Habitat	Size^b^	*N*^c^	Estimate	Standard error	% Coefficient of variation
*Phragmites*	Small	4	0.20	0.14	71
*Phragmites*	Medium	7	0.76	0.12	15
*Phragmites*	Large	3	0.85	0.10	12
*Phragmites*	Extra-large	3	0.86	0.10	11
*Spartina*	Small	14	0.04	0.04	93
*Spartina*	Medium	19	0.33	0.09	27
*Spartina*	Large	15	0.47	0.11	23
*Spartina*	Extra-large	17	0.49	0.11	22

^a^Overdispersion = 2.36

^b^Small = less than 200 g, medium = 200 to 500 g, large = 501 to 1000 g, and extra-large = greater than 1000 g

^c^Total number of carcasses deployed in each habitat and size category

**Fig. 1:**
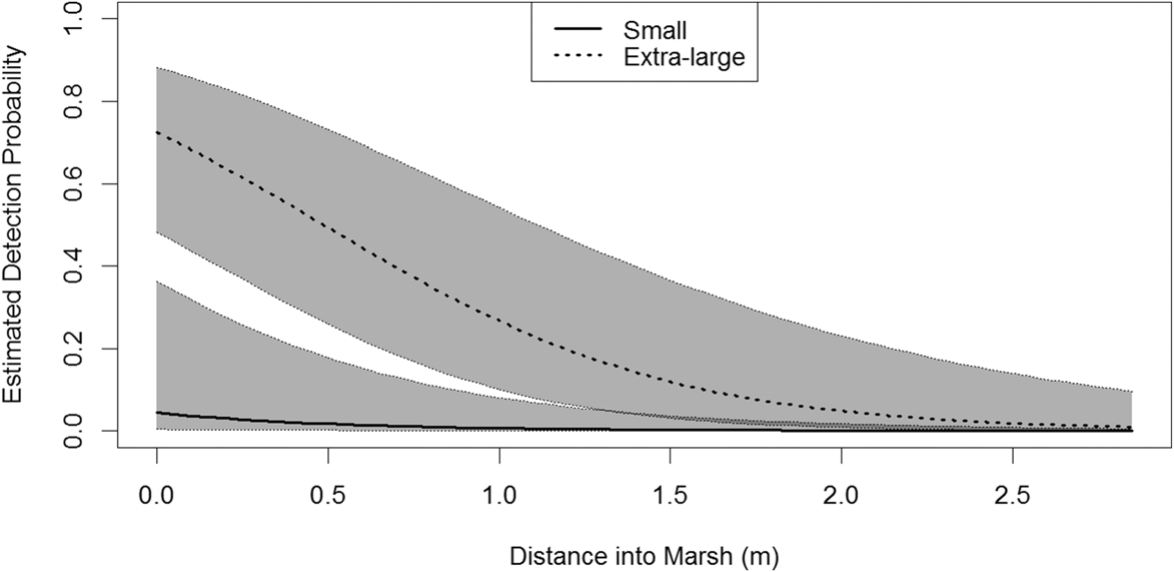
Fitted values comparing expected detection rates as a function of distance into marsh for small (< 200 g) and extra-large (> 1000 g) carcasses in *Spartina*-dominated habitats, Louisiana, 2011

Because the interaction model with categorical size predictor did not converge, we used an interaction model with continuous size predictor (1 = small, 2 = medium, 3 = large, 4 = extra-large) to choose parameters with which to simulate data for the power analyses. We conducted simulations based on models with intercept *β*_intercept_ = − 1, size effect *β*_size_ = 1 (increase per successive size class), and habitat effect *β*_habitat_ = − 2, with and without interaction effect (*β*_size × habitat_ = 0.5 or 0). Statistical power for detecting effects under the unbalanced sampling distribution of 82 carcasses in the marsh edge study design, with 3 searchers per carcass, varied with nearly 100% power for detecting size effects, 94% power for habitat effects, and only 16% power for interaction effects (Fig. 1). Power generally increased with increasing numbers of carcasses, increasing searches per carcass, and with balanced sampling across carcass size and habitat factors. We conducted additional simulations using other parameter values and with overdispersion and found that power of detecting effects generally decreased with absent or decreasing effect sizes and increased with unaccounted overdispersion (Supplemental Materials, Fig. S1).

**Fig. 2:**
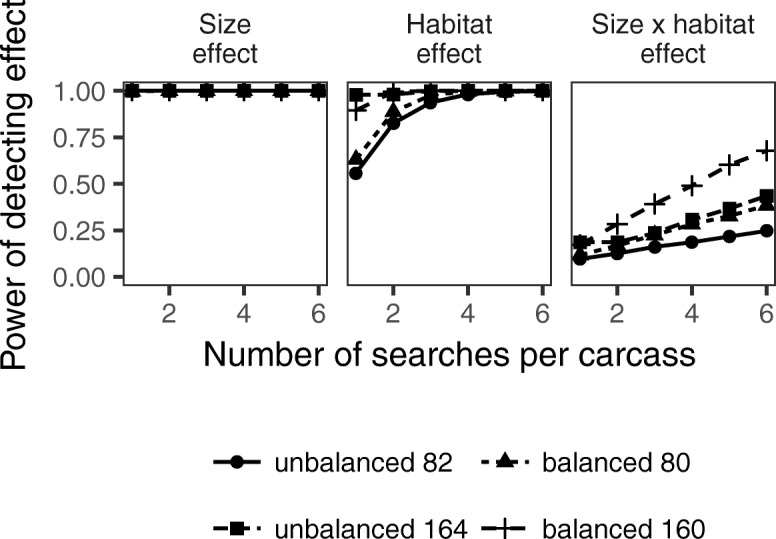
Power of detecting size, habitat type, and size × habitat interaction effects as functions of the number of searches per carcass trial, number of carcass trials, and whether carcass sizes have a balanced or unbalanced distribution among 4 size classes and 2 habitat types. Data were simulated according to a generalized linear model with a continuous size covariate (1 = small, 2 = medium, 3 = large, 4 = extra-large) and model parameters that describe a detection rate which increases with size and is lower for one habitat than the other (*β*_intercept_ = − 1, *β*_size_ = 1, *β*_habitat_ = − 2) and an interaction effect describing mitigated differences between the two habitats as carcass size increases (*β*_size × habitat_ = 0.5), i.e., where habitat differences are greater for small carcasses than for large carcasses, under 4 study designs: 82 carcasses in an unbalanced design similar to the marsh edge study, double-sized (164 carcasses) unbalanced study, and 80 and 160 carcasses in a balanced design with equal carcasses distributed among size and habitat categories. Power was estimated as the proportion of simulated datasets that resulted in significant size, habitat, or interaction effects when tested at the 0.05 significance level

Fifty-five carcasses did not move in the *Spartina*-dominated marshes during the marsh edge study and were included in the analysis of the effect of distance into marsh on detection probability. The $\hat{c}$ estimate based on the global model with distance into marsh, habitat type, and carcass size was 1.58. The best model based on QAIC_*c*_ included an additive effect of distance into marsh and carcass size (Table 3). Detection rates declined with increased distance into the marsh (*β*_distance_ = − 1.98, SE = 0.44; Fig. 2) and increased rapidly from small- to medium-sized carcass, but was similar among medium, large, and extra-large carcasses (*β*_medium_ = 3.78, SE = 1.37; *β*_large_ = 5.59, SE = 1.51; *β*_extra-large_ = 4.04, SE = 1.38).

## Discussion

The need to quantify mortality associated with human activities is an ongoing matter for a variety of species, habitats, and potential sources of mortality (e.g., wind and solar energy, oil spills). A large body of literature exists pertaining to carcass detection probability in terrestrial, non-coastal habitats as part of investigating avian collisions with human-made structures, such as wind turbines, and agricultural pesticide mortality events. Similar to our results for coastal shorelines, these terrestrial studies acknowledge the generally increasing detection probability with carcass size and that detection probability can vary among habitat types (Erickson et al. 2000; Osborn et al. 2000; Stevens et al. 2011; ICF International 2012). Studies associated with the interface between terrestrial habitats and alternative energy attempt to quantify mortality associated with stationary point-source hazards such as wind turbines or solar structures and may involve continuous monitoring of the presence of organisms (bats and birds) in the vicinity of the hazard. Such monitoring is not usually feasible for oil spills, in which the constantly changing and moving oil slick presents a hazard that covers a much larger area than point sources. Nevertheless, our detection probabilities can be informative to studies of bird mortality caused by structures, given similar habitat characteristics, carcass search methods, and other variables. Our analyses provide the first detection probabilities for avian carcasses along coastal shorelines in the northern Gulf of Mexico and can be informative to other oil spill investigations that may occur in that region.

Our detection rate estimates for sandy beaches varied by scavenging state, but were high (i.e., > 0.81) for both heavily and intact/slightly scavenged carcasses. Fowler and Flint (1997) estimated similar detection rates along sandy beaches (from ~ 0.80 to > 0.90) for king eiders (*Somateria spectabilis*) in Alaska and also noted that scavenging reduced detectability. Byrd et al. (2009) observed increased detection rates from approximately 0.40 to 0.70 by having search teams conduct a second survey along the same segment. They suggest that their relatively low detection rate was due to scavenging that reduced the size of carcasses. We corrected for scavenging state and noted that medium-sized carcasses in our study had similar detection rates to Byrd et al. (2009). Ford et al. (2006) also studied detection rates of carcasses along sandy beaches in California and observed lower detection rates than in our study, which we believe were due to differences in survey methods. Searchers walked along beaches in our study whereas searchers scanned for carcasses from motorized vehicles in Ford et al. (2006). Ford et al. (2006) reported that small and large carcasses had a detection rate = 0.13 and 0.44, respectively, when surveys were conducted from all-terrain vehicles, and  0.03 and 0.41, respectively, when surveys were conducted from pick-up trucks. 

Our detection probabilities for carcasses on marsh edges increased from small to medium size, appeared to level off for medium, large and extra-large carcasses, and appeared to be slightly higher in *Phragmites*-dominated marshes compared with *Spartina*-dominated ones. Few other studies have been conducted that evaluate searcher efficiency along edges of coastal or freshwater wetlands, so it is hard to compare our results with others. Ford et al. (unpublished manuscript: https://nrm.dfg.ca.gov/FileHandler.ashx?DocumentID=20097) found the efficiencies of searchers walking through coastal California marshes to be 0.24 for small bird carcasses (< 50 g) and 0.42 for larger carcasses (> 250 g). Our estimates for small birds in *Spartina* are lower (~ 0.04) and for large carcasses in *Phragmites* are considerably greater (> 0.76). Deploying equal numbers of male and female red-winged blackbirds (40–75 g) along the edge of dense cattail marshes in the upper Midwestern US, Linz et al. (1991) found an overall detection rate of 0.81 (SD = 0.11), which is considerably greater than our findings. Their higher estimates are not attributable to using experienced searchers, as our searchers were also experienced, or to coloration, as brightly colored males were found only slightly more often than drab-colored females. Their higher estimates may be due to higher visibility in cattail marshes than coastal marshes but is more likely to be due to differences in survey platform. Searchers from Linz et al. (1991) conducted surveys by foot rather than by boat as occurred in our study.

Previous studies have reported detection rate estimates that differed among habitats, including bay (Ford et al. 2009), rocky shore (Fowler and Flint 1997; Ford et al. 2006), marsh edge (Ford et al. 2006), and sandy beaches (Fowler and Flint 1997, Ford et al. 2006). Our results also indicated habitat differences, because we observed that detection rates along the marsh edge were lower than what we observed for sandy beaches. We also observed that the type of marsh edge can influence detection probability. Overall, detection probability was lower for each size class in *Spartina*-dominated marshes compared with *Phragmites*-dominated ones, which may be due to greater density of vegetation or substrate/marsh coloration making it more difficult to see carcasses. Regardless of carcass size or habitat, carcasses that move into the marsh from the edge are more likely to be missed. We did not feel comfortable making specific estimates of distance-related detection probabilities with our data, given the uncertainty in distance measurements; however, we did observe a strong influence of distance into marsh on detection in *Spartina*-dominated marsh, illustrating the potential importance of controlling for distance to help elucidate patterns with other factors.

We calculated the detection probabilities for various combinations of bird carcass size, scavenging state, and habitat type for coastal shoreline areas. Other factors may influence detection probability, but the field investigators were limited by the carcasses available for use in the field studies, and thus, data on the effect of other factors were not available to be included in these analyses. For example, the field investigators did not have carcasses available to represent the full suite of size classes that are represented in the bird carcasses collected during the spill. The largest carcasses collected during the spill were American white pelicans (*Pelecanus erythrorhynchos*) weighing roughly 7500 g. The largest carcasses used in the sandy beach and marsh edge detection probability studies were herring gulls (*Larus argentatus*) weighing roughly 1000 to 1200 g and brown pelicans (*Pelecanus occidentalis*) weighing roughly 3700 g, respectively. We cannot necessarily presume that the detection rates for carcasses that are larger and smaller than those evaluated in our analyses are always greater and lesser, respectively, than the detection rates estimated by the analyses presented in this document, because our estimates indicate that large and extra-large carcasses appear to have similar detection probabilities and other factors may influence detection, e.g., coloration and attractiveness to scavengers.

We did not evaluate coloration of carcasses and how they contrasted with surroundings. Carcasses with coloration that strongly contrasts with the surrounding environment should be easier to detect. For example, brightly colored, clean (i.e., unscavenged, not covered with mud, sand, or oil) carcasses should be easier to find on beaches with homogeneous dark-colored sediments than drab-colored or unclean carcasses. Similarly, drab-colored carcasses on homogenous white sandy beaches may be easier to detect than white-colored carcasses. Data that would quantify the degree of contrast were not collected during our studies. The majority of our study carcasses (e.g., gulls) would have appeared as combinations of gray, black, and white when deployed for study. The sandy beaches varied in color from dark tan/light brown to light tan/off-white, and the marsh edges had dark-colored water and tan and green vegetation. Approximately two-thirds of the birds impacted by the DWH oil spill (gulls, terns, and northern gannet) (Varela and Martin 2015) had coloration similar to the study carcasses. While there may be a potential for bias in our detection probability estimates due to carcass coloration, the direction and magnitude of such bias, if any, cannot be estimated without additional data that quantifies the amount of contrast between carcasses and their surroundings.

Another factor not evaluated is the effect of natural burial processes on detection probability. Sandy beaches are dynamic systems, with sand being accreted onto and eroded from the beach with seasonal and daily frequencies (e.g., driven by local tides and waves) and with storm events. Marine debris, such as floating vegetation and trash, can also be deposited onto beaches, as well as onto marsh edges. It is possible that carcasses deposited on shorelines could become partially buried by these processes. However, information from the sandy beach carcass persistence study (IEc 2011, Varela and Zimmerman 2020), the marsh edge carcass persistence study (Trustee Bird Technical Working Group 2012; Varela and Zimmerman 2020), and a study to evaluate the probability that bird carcasses drifting in nearshore waters would strand on shorelines in the northern Gulf of Mexico (Martin et al. 2020) suggested that natural burial of carcasses rarely occurred during the summer and autumn of 2011 in the northern Gulf of Mexico. Partial burial could potentially reduce detectability by decreasing the contrast of birds with the surrounding environment. Therefore, the field investigators in the sandy beach detection probability study did occasionally place some sand or marine debris on top of placed study carcasses, representing a partial burial, but quantitative data on the degree of burial were not collected. As detection probability is the probability of finding carcasses that are available for detection, a completely buried carcass would be temporarily or permanently not available for detection (and thus would be accounted for in mortality models by carcass persistence rates).

Lastly, our results for sandy beaches may be biased high by some unknown amount due to the inability to completely hide the footprints of carcass deployment personnel. Attempts were made to minimize footprints and tracks that might provide clues to searchers on all beaches. On some beaches, particularly the remote beaches seldom visited by people, recent rains or blowing sand cleaned the beach of tracks before the carcass deployment personnel arrived, and thus, their footprints were the only footprints on those beaches when searchers conducted their surveys. During the peer-review of this manuscript, a reviewer suggested using unmanned aircraft systems (UAS, a.k.a., drones) to deposit carcasses, which would eliminate a potential bias associated with footprints from researchers. Although we found this an interesting suggestion, it may not be feasible for several reasons, such as weight-carrying limitations of the available UAS with respect to larger bird carcasses, complications with temporary airspace closures typically implemented by the Federal Aviation Administration during oil spills, potential damage to UAS from entraining beach sand into motors and other moving parts if flown too close to the ground, potential of UAS prop wash to cause a “UAS footprint” on the shoreline, and increased cost of study implementation. In addition, regulatory requirements may exist for minimum altitudes above and distances from sensitive wildlife that may be present along the shorelines of the study area, which could potentially prohibit researchers from flying drones sufficiently close to the ground to place study carcasses in a controlled manner. However, the UAS technology and the list of tested and endorsed protocols for flying around wildlife continue to develop, so using UAS could be an option for future studies.

The field investigators originally intended only to estimate carcass size–specific detection rate for a model specific to estimating avian acute mortality associated with the DWH oil spill along the northern coast of the Gulf of Mexico. They noted that other factors could influence detection of bird carcasses along shorelines (i.e., carcass scavenging state, position on the beach, marsh habitat, distance into marshes) and deployed carcasses in variations of these factors (e.g., three positions on the beach). The studies were not originally designed to quantify the influence of each of the factors on detection probability and thus, sample distributions were not ideal for that purpose. However, we saw an opportunity to explore the influence of these factors on detection probability using subsets of the available data. A lack of prior information on detection rates specific to the study area and limited availability of carcasses for certain size classes reduced our ability to assess how carcass size might interact with other factors. Nonetheless, we attempted to explore these relationships by estimating models that considered additive and interaction effects of these factors.

We conducted simulation analyses to determine the power of the marsh edge study design to identify effects on detection rate estimates, with the goal of sharing insights on study design considerations for future investigations of detection probability. Such effects, when accurately determined, can help to refine estimates of carcass size–specific detection rates. Our power analyses assessed the probability of finding significant effects on detection rates, under the design and sample size of the marsh edge study. We found that the power of detecting size and marsh type effects was large (> 90%), but the power of detecting interaction effects was low (16%). We also found that the power to identify an interaction effect of carcass size and marsh habitat type on detection probability increases with increasing numbers of study carcasses, with increasing numbers of search trials per carcass, and with more balanced sampling distributions across predictor variables. We suggest using a sampling design with balanced samples of sufficient size in each of the combinations of the predictor variables to most effectively improve the ability to detect an interaction effect. However, if the availability of study carcasses is limiting, increasing numbers of search trials for each carcass can also improve power, but to a lesser extent.

## Conclusion

Wildlife biologists have known about bias due to imperfect detection for decades (see summaries in Otis et al. 1978, Thompson et al. 1998, and Anderson 2001). Counts of birds or their carcasses that are not adjusted for imperfect detection yield indices that, in some cases, may correlate with abundance of the birds/carcasses in the survey area, which may be acceptable in some situations (Johnson 2008). However, the absolute number and associated estimates of uncertainty, as opposed to an index representing a proportion of the bird/carcass population, are important when developing a legal claim for natural resource damages under the Oil Pollution Act (33 U.S.C. 2701 et seq.). Our study demonstrates that, even when carcasses are present and visible, detection rates are not 100% and may be particularly low in some habitats and for some carcasses. Conducting intensive studies such as ours is not always feasible for each oil spill and understanding specific factors that may correlate with detection rates can be useful in other assessments (White 2005). We observed that detection rates can be extremely variable and depend on a variety of factors including the size of the carcass, habitat type, and carcass scavenged state. If biologists do not account for these factors when developing acute mortality models, the resulting mortality estimates can be unnecessarily biased. Therefore, we suggest that biologists test detection probability under local conditions when implementing beached bird monitoring programs when resources are available to do so. Power analyses can be used to make planning decisions about sample size of carcasses, number of search trials or search teams, and the relative allocation of samples among factors to improve inferences in the face of logistical constraints to implementing searcher efficiency studies.

## Electronic supplementary material

ESM 1 (PDF 240 kb)
