# Supplementary material for: Detection probabilities of bird carcasses along sandy beaches and marsh edges in the northern Gulf of Mexico
Source: Environ Monit Assess. 2020 Mar 17;191(Suppl 4):816. doi: 10.1007/s10661-019-7924-z (PMC7078141; doi:10.1007/s10661-019-7924-z)
Supplement: Supplementary file 1 — (PDF 240 kb) [file 10661_2019_7924_MOESM1_ESM.pdf]

Supplementary material

Article title: Detection probabilities of bird carcasses along sandy beaches and marsh edges in the northern Gulf of Mexico

Journal name: Environmental Monitoring and Assessment

Authors: Guthrie S. Zimmerman, Veronica W. Varela, Julie L. Yee

Corresponding author: Guthrie S. Zimmerman, USFWS, guthrie_zimmerman@fws.gov

Fig S1 Power of detecting size × habitat interaction effects and probability of separation in the data, i.e. where all carcass searches for at least one combination of size and habitat class resulted in the same outcome, as functions of the number of search trials per carcass, number of carcasses, and whether carcass sizes have a balanced or unbalanced distribution among 4 size classes and 2 habitat types. Data were simulated according to a binomial distribution and generalized linear model with a continuous size covariate (1=small, 2=medium, 3=large, 4=extra-large) and model parameters that describe a detection rate which increases with size and is lower for one habitat than the other (βintercept = -1, βsize = 1, βhabitat = –2), with 3 variations: no interaction effect with no overdispersion, with interaction effect (βsize x hab = 0.5) and no overdispersion, and with interaction and overdispersion factor 2.35. Data were simulated under 4 study designs: 82 carcasses in an unbalanced design similar to the marsh edge study (82 carcasses), double sized unbalanced study (164 carcasses), and 80 and 160 carcasses in a balanced design with equal carcasses distributed among size and habitat classes. Power was estimated as the proportion of 10,000 simulated datasets that resulted in a significant interaction effects when tested at the 0.05 significance level, and probability of separation was estimated as the proportion of 10,000 simulated datasets with separation in the data.

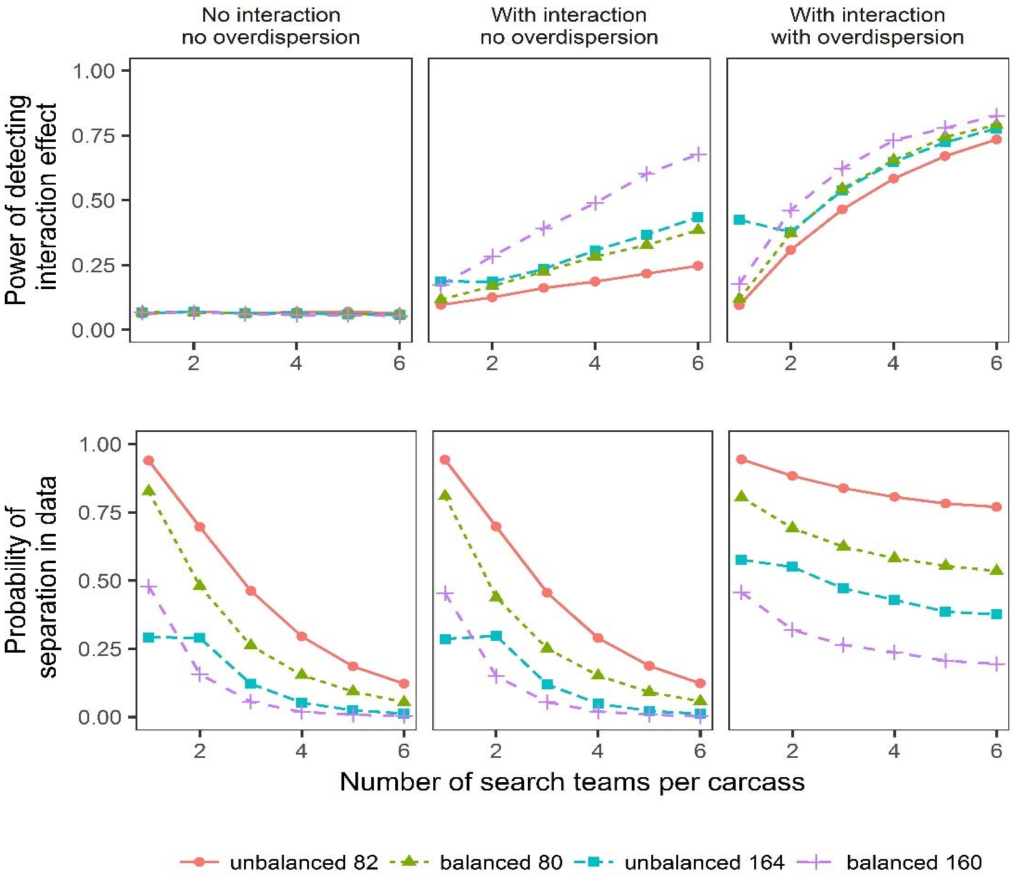

Supplementary material

Article title: Detection probabilities of bird carcasses along sandy beaches and marsh edges in the northern Gulf of Mexico
Journal name: Environmental Monitoring and Assessment
Authors: Guthrie S. Zimmerman, Veronica W. Varela, Julie L. Yee
Corresponding author: Guthrie S. Zimmerman, USFWS, guthrie_zimmerman@fws.gov

Table description

Results of simulation analyses to calculate power of detecting size, habitat, and size by habitat interaction effects for 4 study designs. Each row summarizes the outcomes of a set of 10,000 simulated datasets and their corresponding model results. Each dataset was simulated according to a binomial distribution and generalized linear model with a continuous size covariate (1=small, 2=medium, 3=large, 4=extra-large) and model parameters that describe a detection rate which increases with size and is lower for one habitat than the other (βintercept = -1, βsize = 1, βhabitat = –2). Sets of simulations were repeated and varied by overdispersion factor, number of search trials per carcass (1 through 6), and presence of interaction effect (βsize x hab = 0.5). Results included mean estimated overdispersion, probability of separation in the data, and powers of detecting size, habitat, and size x habitat interaction effects.

Table fields

od: prescribed overdispersion factor for a 3-search study (1=not overdispersed; 2.35=overdispersed similar to marsh edge study

study_design: unbalanced sample of 82 carcasses based on marsh edge study, unbalanced sample of 164 carcasses based on double-sized marsh edge study, balanced sample of 80 carcasses based on 10 carcasses per combination of 4 size classes and 2 habitat types, and 160 carcasses based on double-sized balanced study

n_trials: number of trials per carcass (1 through 6)

szxhab: size by habitat interaction parameter (0=no interaction; 0.5=interaction)

mean_OD: mean OD estimate averaged across 10,000 simulations

prob_sep: probability of separation in the data, i.e. where all carcass searches for at least one combination of size and habitat class resulted in the same outcome

power_sz: power of detecting size effect

power_hab: power of detecting habitat effect

power_szxhab: power of detecting size x habitat interaction effect

od	study_design	n_trials	szxhab	mean_OD	prob_sep	power_sz	power_hab	power_szxhab
1	unbalanced 82	1	0	NA	0.94	0.93	0.88	0.06
1	unbalanced 82	1	0.5	NA	0.94	1.00	0.56	0.10
1	unbalanced 82	2	0	0.98	0.70	1.00	0.99	0.07
1	unbalanced 82	2	0.5	0.98	0.70	1.00	0.83	0.13
1	unbalanced 82	3	0	1.00	0.46	1.00	1.00	0.06
1	unbalanced 82	3	0.5	1.00	0.46	1.00	0.94	0.16
1	unbalanced 82	4	0	1.00	0.30	1.00	1.00	0.07
1	unbalanced 82	4	0.5	1.00	0.29	1.00	0.98	0.19
1	unbalanced 82	5	0	1.00	0.19	1.00	1.00	0.07
1	unbalanced 82	5	0.5	1.01	0.19	1.00	0.99	0.22
1	unbalanced 82	6	0	1.01	0.12	1.00	1.00	0.06
1	unbalanced 82	6	0.5	1.01	0.12	1.00	1.00	0.25
1	unbalanced 164	1	0	NA	0.29	1.00	1.00	0.07
1	unbalanced 164	1	0.5	NA	0.29	1.00	0.98	0.19
1	unbalanced 164	2	0	1.00	0.29	1.00	1.00	0.07
1	unbalanced 164	2	0.5	1.00	0.30	1.00	0.98	0.19
1	unbalanced 164	3	0	1.01	0.12	1.00	1.00	0.06
1	unbalanced 164	3	0.5	1.01	0.12	1.00	1.00	0.24
1	unbalanced 164	4	0	1.01	0.05	1.00	1.00	0.06
1	unbalanced 164	4	0.5	1.01	0.05	1.00	1.00	0.31
1	unbalanced 164	5	0	1.01	0.02	1.00	1.00	0.06
1	unbalanced 164	5	0.5	1.01	0.02	1.00	1.00	0.37
1	unbalanced 164	6	0	1.01	0.01	1.00	1.00	0.06
1	unbalanced 164	6	0.5	1.01	0.01	1.00	1.00	0.43
1	balanced 80	1	0	NA	0.83	0.96	0.97	0.07
1	balanced 80	1	0.5	NA	0.81	1.00	0.63	0.12
1	balanced 80	2	0	0.98	0.48	1.00	1.00	0.07
1	balanced 80	2	0.5	0.99	0.44	1.00	0.89	0.17
1	balanced 80	3	0	1.00	0.26	1.00	1.00	0.06
1	balanced 80	3	0.5	1.00	0.25	1.00	0.98	0.22
1	balanced 80	4	0	1.01	0.15	1.00	1.00	0.06
1	balanced 80	4	0.5	1.00	0.15	1.00	1.00	0.28
1	balanced 80	5	0	1.01	0.09	1.00	1.00	0.06
1	balanced 80	5	0.5	1.01	0.09	1.00	1.00	0.33
1	balanced 80	6	0	1.01	0.05	1.00	1.00	0.06
1	balanced 80	6	0.5	1.01	0.06	1.00	1.00	0.38
1	balanced 160	1	0	NA	0.48	1.00	1.00	0.07
1	balanced 160	1	0.5	NA	0.45	1.00	0.89	0.17
1	balanced 160	2	0	1.01	0.16	1.00	1.00	0.07
1	balanced 160	2	0.5	1.01	0.15	1.00	0.99	0.28
1	balanced 160	3	0	1.01	0.06	1.00	1.00	0.06
1	balanced 160	3	0.5	1.01	0.05	1.00	1.00	0.39
1	balanced 160	4	0	1.01	0.02	1.00	1.00	0.06
1	balanced 160	4	0.5	1.01	0.02	1.00	1.00	0.49
1	balanced 160	5	0	1.01	0.01	1.00	1.00	0.06
1	balanced 160	5	0.5	1.01	0.01	1.00	1.00	0.60
1	balanced 160	6	0	1.01	0.00	1.00	1.00	0.05
1	balanced 160	6	0.5	1.01	0.00	1.00	1.00	0.68

od	study_design	n_trials	szxhab	mean_OD	prob_sep	power_sz	power_hab	power_szxhab
2.35	unbalanced 82	1	0	NA	0.95	0.93	0.88	0.06
2.35	unbalanced 82	1	0.5	NA	0.94	1.00	0.56	0.10
2.35	unbalanced 82	2	0	1.59	0.89	1.00	0.97	0.26
2.35	unbalanced 82	2	0.5	1.60	0.88	1.00	0.76	0.31
2.35	unbalanced 82	3	0	2.24	0.84	1.00	0.99	0.42
2.35	unbalanced 82	3	0.5	2.26	0.84	1.00	0.84	0.46
2.35	unbalanced 82	4	0	2.89	0.81	1.00	0.99	0.56
2.35	unbalanced 82	4	0.5	2.92	0.81	1.00	0.89	0.58
2.35	unbalanced 82	5	0	3.54	0.80	1.00	0.99	0.66
2.35	unbalanced 82	5	0.5	3.57	0.78	1.00	0.90	0.67
2.35	unbalanced 82	6	0	4.20	0.77	1.00	1.00	0.73
2.35	unbalanced 82	6	0.5	4.23	0.77	1.00	0.92	0.73
2.35	unbalanced 164	1	0	NA	0.57	1.00	1.00	0.34
2.35	unbalanced 164	1	0.5	NA	0.58	1.00	0.92	0.42
2.35	unbalanced 164	2	0	1.66	0.56	1.00	1.00	0.26
2.35	unbalanced 164	2	0.5	1.66	0.55	1.00	0.94	0.38
2.35	unbalanced 164	3	0	2.34	0.47	1.00	1.00	0.43
2.35	unbalanced 164	3	0.5	2.35	0.47	1.00	0.97	0.54
2.35	unbalanced 164	4	0	3.02	0.43	1.00	1.00	0.56
2.35	unbalanced 164	4	0.5	3.03	0.43	1.00	0.98	0.65
2.35	unbalanced 164	5	0	3.71	0.40	1.00	1.00	0.65
2.35	unbalanced 164	5	0.5	3.72	0.39	1.00	0.99	0.72
2.35	unbalanced 164	6	0	4.39	0.38	1.00	1.00	0.71
2.35	unbalanced 164	6	0.5	4.39	0.38	1.00	0.99	0.78
2.35	balanced 80	1	0	NA	0.83	0.96	0.97	0.06
2.35	balanced 80	1	0.5	NA	0.81	1.00	0.63	0.12
2.35	balanced 80	2	0	1.59	0.71	1.00	1.00	0.26
2.35	balanced 80	2	0.5	1.60	0.69	1.00	0.84	0.37
2.35	balanced 80	3	0	2.25	0.65	1.00	1.00	0.42
2.35	balanced 80	3	0.5	2.26	0.62	1.00	0.90	0.54
2.35	balanced 80	4	0	2.90	0.61	1.00	1.00	0.55
2.35	balanced 80	4	0.5	2.93	0.58	1.00	0.93	0.65
2.35	balanced 80	5	0	3.56	0.58	1.00	1.00	0.64
2.35	balanced 80	5	0.5	3.59	0.55	1.00	0.94	0.74
2.35	balanced 80	6	0	4.22	0.55	1.00	1.00	0.70
2.35	balanced 80	6	0.5	4.24	0.53	1.00	0.96	0.79
2.35	balanced 160	1	0	NA	0.47	1.00	1.00	0.07
2.35	balanced 160	1	0.5	NA	0.46	1.00	0.89	0.18
2.35	balanced 160	2	0	1.67	0.35	1.00	1.00	0.26
2.35	balanced 160	2	0.5	1.68	0.32	1.00	0.97	0.46
2.35	balanced 160	3	0	2.35	0.28	1.00	1.00	0.41
2.35	balanced 160	3	0.5	2.36	0.26	1.00	0.99	0.62
2.35	balanced 160	4	0	3.04	0.24	1.00	1.00	0.53
2.35	balanced 160	4	0.5	3.04	0.24	1.00	0.99	0.73
2.35	balanced 160	5	0	3.72	0.22	1.00	1.00	0.61
2.35	balanced 160	5	0.5	3.74	0.21	1.00	1.00	0.78
2.35	balanced 160	6	0	4.40	0.20	1.00	1.00	0.67
2.35	balanced 160	6	0.5	4.42	0.19	1.00	1.00	0.83
